# Spatiotemporal Rank Filtering Improves Image Quality Compared to Frame Averaging in 2-Photon Laser Scanning Microscopy

**DOI:** 10.1371/journal.pone.0150430

**Published:** 2016-03-03

**Authors:** Henry Pinkard, Kaitlin Corbin, Matthew F. Krummel

**Affiliations:** 1 Department of Pathology, University of California San Francisco, San Francisco, California, United States of America; 2 Biological Imaging Development Center, University of California San Francisco, San Francisco, California, United States of America; 3 Computational Biology Graduate Group, University of California Berkeley, Berkeley, California, United States of America; Glasgow University, UNITED KINGDOM

## Abstract

Live imaging of biological specimens using optical microscopy is limited by tradeoffs between spatial and temporal resolution, depth into intact samples, and phototoxicity. Two-photon laser scanning microscopy (2P-LSM), the gold standard for imaging turbid samples *in vivo*, has conventionally constructed images with sufficient signal-to-noise ratio (SNR) generated by sequential raster scans of the focal plane and temporal integration of the collected signals. Here, we describe spatiotemporal rank filtering, a nonlinear alternative to temporal integration, which makes more efficient use of collected photons by selectively reducing noise in 2P-LSM images during acquisition. This results in much higher SNR while preserving image edges and fine details. Practically, this allows for at least a four fold decrease in collection times, a substantial improvement for time-course imaging in biological systems.

## Introduction

All modalities of fluorescence microscopy face a practical limit to the number of photons they can collect from a given sample, and must make tradeoffs within this “photon budget” among spatial and temporal resolution, photodamage, and depth of imaging. Two-photon laser scanning microscopy (2P-LSM) [1] has enabled imaging of the behavior of mammalian cells *in vivo* [2] and is the method of choice for live imaging deep into scattering tissues including lymph nodes, lung, pancreas, and brain. The photon budget in 2P-LSM *in vivo* is an especially important consideration since photodamage, which compromises viability and can alter cell behavior, responds to increases in excitation power at higher order (i.e. supra-quadratic) rates compared to fluorescence emission [3,4]. As a result, increasing excitation laser intensity is not always a viable strategy for collecting enough photons to provide images with a sufficient signal to noise ratio for visualization and analysis. Many experiments are instead forced to rely upon averaging (or equivalently, integrating) repeated scans of the same area; however, this leads to a loss of temporal resolution and can introduce blur artifacts of dynamic processes [5]. Increasing the speed of imaging without compromising viability is an essential challenge that must be overcome to tap the full potential of 2P-LSM to observe the complex spatiotemporal behavior of biological circuits. A number of strategies have been developed to address this problem, including spatial or temporal multiplexing of excitation and intelligent spatial modulation of laser intensity based on counts of collected photons, but each has its drawbacks [6]. Improvements in the design of high numerical aperture objective lenses and high quantum yield fluorescent proteins will also surely yield progress in this area. Still, maximizing the information extracted from a finite number of photons in 2P-LSM remains an open challenge.

One promising area for addressing this challenge lies in image filtering and processing, and in particular their use in combination at acquisition time [5]. Filtering methods already find widespread use in 2P-LSM in two stages: frame averaging that takes place at acquisition time (equivalent to a linear, low-pass filter) followed by post-acquisition Gaussian (linear, low-pass) or median (nonlinear) filtering [7–9]. More complex de-noising algorithms have outperformed temporal averaging for the specialized case of calcium fluxes in a single focal plane [10,11] but rely on parametric models of signal and noise and have not been shown to be effective for de-noising 3D volumes over which parameters may vary. Thus, filtering strategies that can make similar improvements and can be applied over three-dimensional volumes have great promise for improving performance in 2P-LSM.

Given that photons are the limited quantity in biological imaging, an approach to filtering essentially relies on removing a portion of the noise in the SNR equation. Although the probability distributions of noise across the range of hardware implementations of 2-photon microscopes is unknown, a few generalizations can be made about noise in 2P-LSM. Its sources can be broadly grouped into two categories: First, the hardware of excitation and detection such as photomultiplier tubes, the electronics of the analog to digital conversion process, and fluctuations in modulation of excitation beam intensity; Second, the Poisson (shot) noise inherent in photon emission from excited fluorophores. The former includes unwanted fluorescence from regions closer to the sample surface when using high excitation power to image deep into intact samples, stray photons from ambient light, thermally emitted electrons within photomultiplier tubes, or noise in the analog to digital conversion process [12,13]. None of these processes are spatially correlated with locations on the resulting image, giving them the characteristics of digital impulsive (a.k.a. “salt and pepper”) noise, with a duration in time or space of only one pixel [14]. The other major source, shot noise from excited fluorophores, depends upon the strength of the signal [13].

Neither of these types of noise follows a Gaussian distribution, for which linear filters are optimal, and the performance of linear filters is degraded by a single outlier, preventing them from effectively filtering out background noise in 2P-LSM images [15]. When applied spatially, they blur edges and fine details, which can lead to loss of resolution and allow the persistence of the low spatial frequency components of noise [16,17]. Given these shortcomings, nonlinear filters, which have been shown to outperform averaging in medical imaging applications such as ultrasound [18], may be a better choice. Nonlinear filters come in diverse forms with varying complexity [19]. Here we focus on the rank filter (a.k.a. the percentile filter), a relatively simple nonlinear filter implementation with a history of use in processing digital images from other imaging modalities [20–26].

Rank filters take a set of n input values, sort them, and take the output value as the k^th^ value. Rank filters have a single parameter between 0 and 1 that determines the value of k for a given n. For example, a rank of 1 takes the maximum of the set of input values. A rank of 0.5 represents a median filter. Rank filters can effectively eliminate the impulsive noise seen in 2P-LSM since they limit the effect of single input values on signal estimation [14,25]. Furthermore, they do not universally remove high spatial frequencies, allowing them to preserve sharp edges and other details important to human perception and automated object detection [15,27].

The performance of rank filters can be further improved by combining the temporal filtering that occurs at acquisition time and the post-acquisition spatial filtering into a single step (a spatiotemporal rank filter). This filter takes advantage of a reduction in variance that occurs when combining sequential rank filters into a single step and obviates the need for post acquisition processing to improve image quality [15]. Here, we demonstrate the superiority of spatiotemporal rank filtering to frame averaging and characterize its performance so as to provide practical recommendations for its implementation. Given the need to minimize excitation power doses in 2P-LSM and the overall low photon yield, we focus on images with low levels of fluorescence signal.

## Results

**Fig 1:**
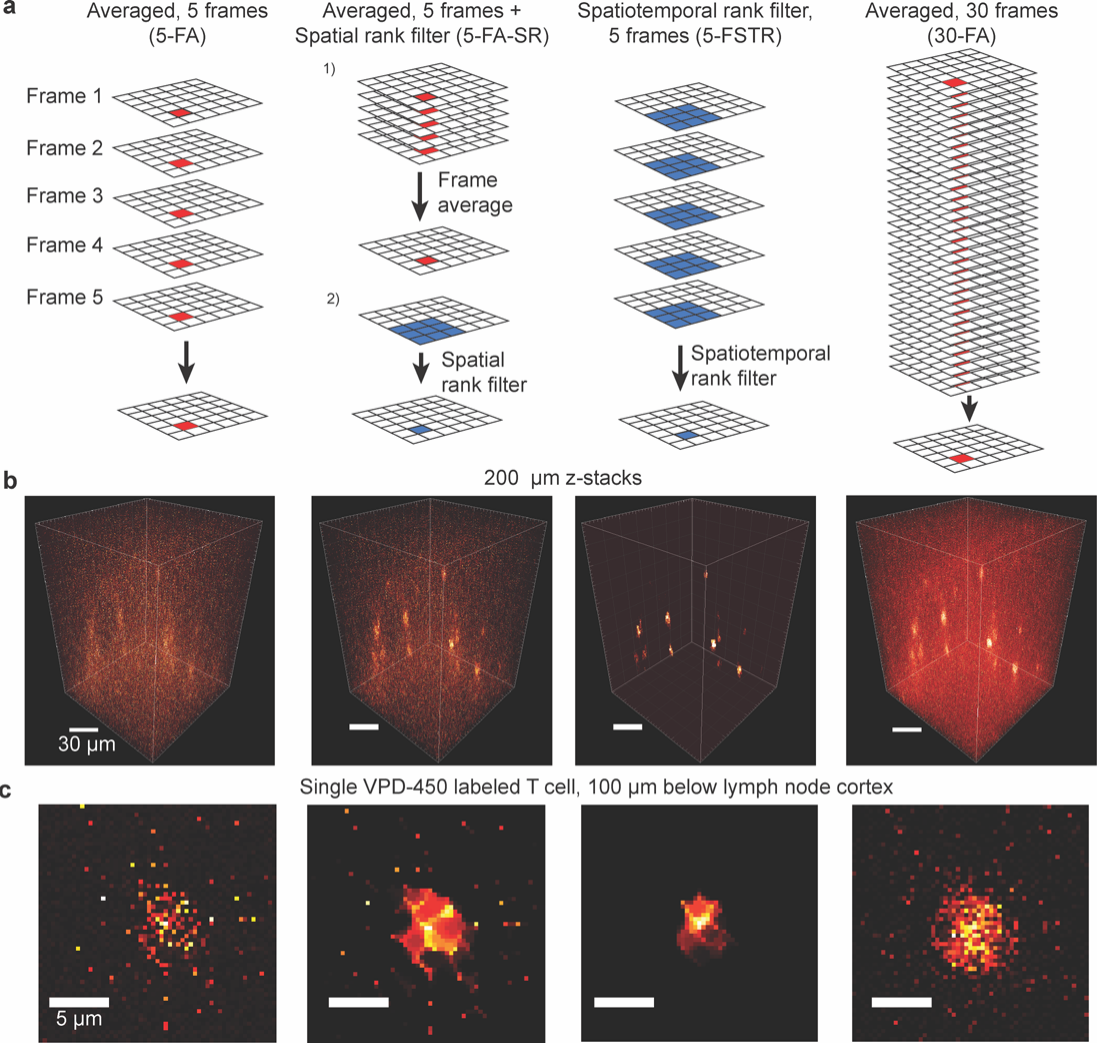
Comparison of frame averaging and rank filtering on VPD-450 labeled T cells within a lymph node. (A) Schematic of various filtering methods. (B) 200 μm z-stacks created by (left-right) frame averaging (5-FA), frame averaging followed by spatial rank filtering with 0.7 μm (2 pixel) radius (5-FA-SR), spatiotemporal rank filtering with 0.7 μm (2 pixel) radius (5-FSTR), frame averaging with 6x as many frames (30-FA). Maximum contrast is set to 0.5 x the maximum pixel value in each image to show cells clearly against background. (C) 2D image of a single T cell 100 μm below the lymph node capsule

To demonstrate the utility of spatiotemporal rank filtering for 3D imaging of living biological tissue, we collected a z-stack extending 200 μm down from the cortex of a mouse lymph node containing T cells labeled with VPD-450, a typical chemical dye used in imaging studies. We initially collected 30 frames at each slice, saved individually, so that we could compare on the same data 5-frame averaging (5-FA), 5-frame averaging followed by spatial rank filtering (5-FA-SR), 5-frame spatiotemporal rank filtering (5-FSTR), which performs the rank on an n by n array of adjacent pixels taken from all 5 frames, and a higher quality frame averaging image with 6x as many frames (i.e. 30) representing an outermost practical limit of frame-averaging (30-FA) (**Fig 1A**). Individual cells were difficult to distinguish in 5-FA images due to high background (**Fig 1B**). 5-FA-SR improved the quality of the image compared to 5-FA, but was unable to eliminate impulsive noise and introduced artifacts in the form of protrusions from the cell that were not present in the 30-FA image. In contrast, the 5-FSTR image was brighter, free of impulsive background noise, and possessed steep gradients delineating the cell edge, even compared to the 30-FA image (**Fig 1C).** However, parts of the cell border in the 5-FSTR image appear to have shrunk compared to the 30-FA image. Although the 5-FSTR was able to recover a high quality image from a much smaller number of frames compared to frame averaging, the small sample of frames in the approach increased the probability of stochastic fluctuations in signal that dip below a level distinguishable from noise.

**Fig 2:**
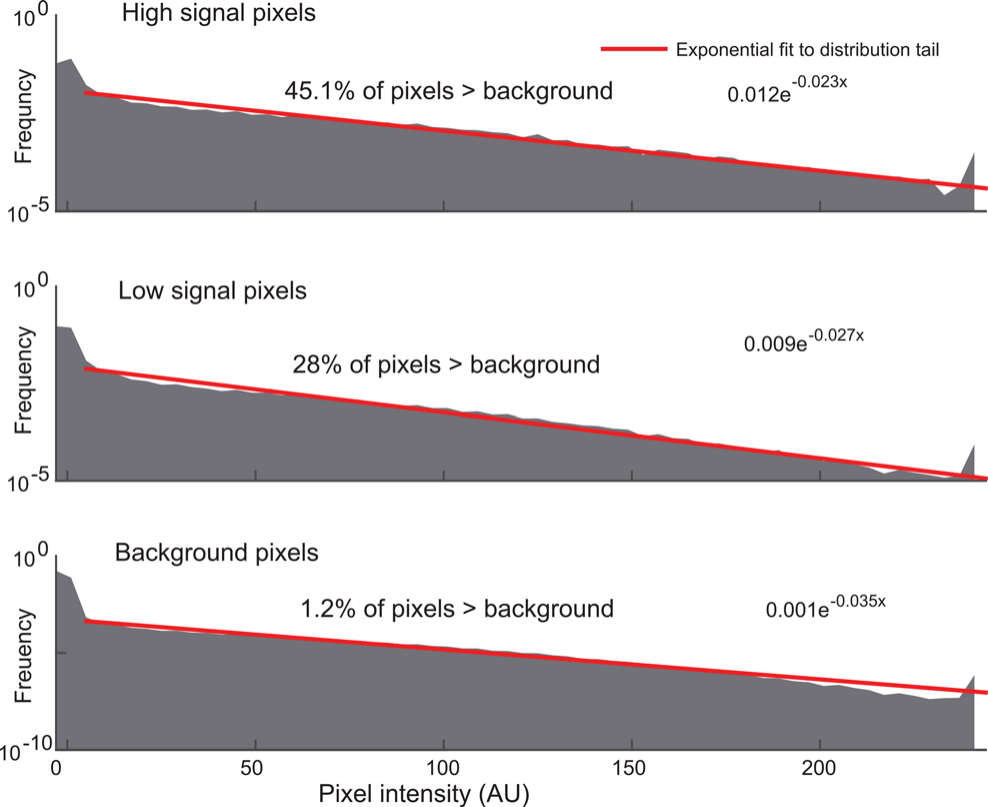
Pixel intensity value distributions at high, low, and no signal regions of bead images. High, low, and no signal regions of fluorescent beads masked to generate empirical distributions of intensity values. Red line indicates exponential fit to distribution tail.

In order to verify the suitability of rank filtering on the basis of the empirical distributions of pixel intensity values in a typical photon-limited biological image, we took a 1000 time point image of 175 nm PS-Speck beads with excitation power low enough that individual beads were indistinguishable from noise unless 5–10 frames were averaged together. We background subtracted these images using the 50^th^ percentile value of a dark image with no excitation power. We then averaged all 1000 frames, and placed masks for pixels of different average intensities or non-bead pixels and assessed histograms of pixel intensity counts (**Fig 2**). We found that background pixels, pixels on parts of beads with low average intensity, and pixels of high average intensity shared a similar empirical distribution function, with a large portion of intensity values indistinguishable from background and tails that decayed close to, but slightly faster than, exponential distributions. These findings provide empirical justification for the use of rank filters, since heavier tailed distributions, such as exponential, have been found to represent best use cases [25].

**Fig 3:**
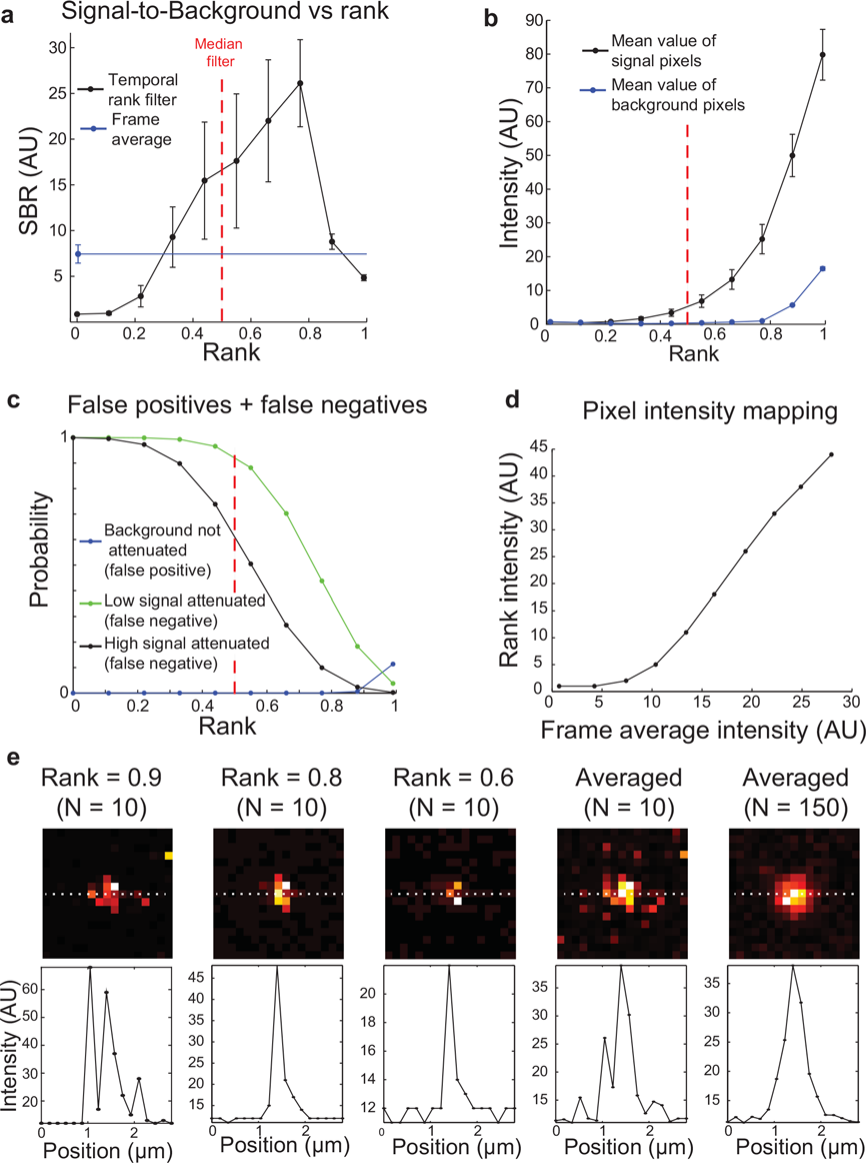
Performance of rank filters on bead images with different rank parameter values. (A) Averaged signal to background of low average intensity pixels when rank filtered with various ranks. Error bars indicate standard deviation. (B) Mean values of bead pixels and background pixels used to generate (A). (C) Probability of attenuating high or low signal (false negative) or not attenuating background (false positive) based on binomial distribution cumulative distribution functions (N = 10) and the proportion of background pixels shown in Fig 2. (D) Mapping of intensity values corresponding to different average values compared to rank filter values. (E) Representative images of single sub-resolution beads and pixel linescans across them showing the failure to attenuate background when the rank parameter is set two high and the loss of dim pixels at the edges of the PSF when rank is set too low. Beads are 170 nm in diameter; pixels are 175 nm; theoretical Abbe resolution of the imaging system is 410 nm.

The use of a rank besides 50% (the median) is an important part of this approach and allows for the filter to adapt to a high noise/low signal environment typical of photon-limited biological imaging. We thus sought to characterize how much the choice of the rank parameter affected detection of signal. For this, we used similar samples as Fig 2 and applied pixel masks to areas without beads and pixel masks for beads with low levels of signal. We then randomly selected sets of 10 consecutive frames to which we applied temporal rank filters (i.e. a rank filter with no spatial component) with varying ranks. We then calculated a signal-to-background ratio (SBR) as the average value of pixels lying within the weak signal mask divided by the average value of pixels within the non-bead mask (**Fig 3A**). This indicates that for 10 serial scans (a typical value for recovering weak signals on our system), temporal rank filtering is capable of outperforming frame averaging by a factor of 3–4.

The use of higher rank values produces increasingly bright and sharp images, especially in areas corresponding to weaker signals, up to a certain point at which image quality rapidly degrades. This drop-off coincides with the point at which the rank filter fails to eliminate high background pixels (**Fig 3B**). However, the increases in average signal seen with increasing rank are not uniform across all signal values. Using the measurements of the proportion of pixels that are indistinguishable from background in **Fig 2**, we calculated the probability of false positives (i.e. noise that the rank filter fails to eliminate) and false negatives (i.e. signal that the rank filter attenuates) for a set of samples of fixed size. The probability of false positives increased with rank, while the probability of false negatives decreased with rank and strength of signal (**Fig 3C**). Thus, the selection of a rank that is low (such as the median filter) results in the attenuation of weak signals, while picking a rank that is too high fails to eliminate background.

Next, we sought to determine the relationship between intensity values generated from rank filter versus those from frame averaging. When using the optimal rank from **Fig 3A**, we again found that rank filtering tends to suppress weaker pixels values—including background and pixels corresponding to signal levels just above background (**Fig 3D**). For medium to strong levels of signal, rank filtered values and frame-averaged values had an approximately linear relationship, though it is worth noting that the slope of this relationship is greater than 1, reflecting the fact that the frame average and the rank filter are estimators of the intensity distribution mean and characteristics of the distribution tail, respectively [15,25]. However, the relationship between the two is monotonic across all intensity values, indicating that, like frame averaged pixel values, higher rank filtered values correspond to higher fluorescence intensity. Unlike the case of frame averaging, these values cannot be assumed to be proportional to the concentration of fluorescent molecules [28]. The images of rank filtered beads (**Fig 3E)** demonstrated that selecting a rank (0.9) that is too high enhanced impulsive background noise, while selecting a rank that is too low (0.6) led the loss of dimmer pixels on the edges of the bead point spread function (PSF). However, with an appropriate choice of rank (0.8), the central peak of the system’s PSF as seen in the 150x frame averaged image was captured while background noise was still suppressed.

**Fig 4:**
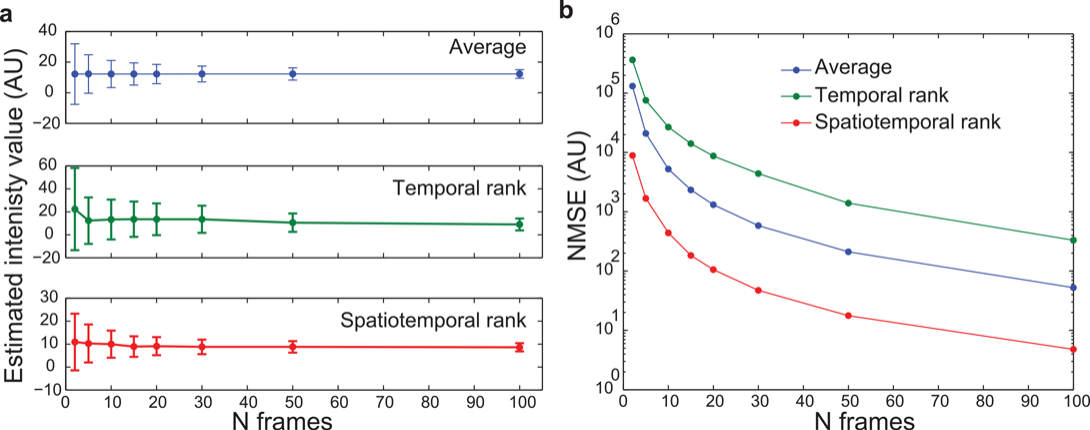
Characterization of average, temporal rank, and spatiotemporal (3x3 pixel) rank estimators. (A) Means of sampling distributions generated from dim signal pixels in fluorescent bead images. Error bars indicate standard deviation (B) Normalized mean squared error

Although rank filtering is clearly capable of producing images with better signal to background ratios, the higher standard deviation of this measurement for rank filtering compared to frame averaging (**Fig 3A**) suggests that pixel intensity estimates for rank filtering might exhibit greater variation. To examine this more closely and discern the effect of sample size on the normalized mean squared error (NMSE) of these estimators, we employed the bootstrap method. Resampling from the distribution of pixel values at spatial locations corresponding to weak signal on the 150x frame averaged image, we generated the mean, variance, and normalized mean squared error of sampling distributions. This analysis showed a slight bias for rank filter estimates with small n (**Fig 4A**), corresponding to a higher value of NMSE compared to frame averaging (**Fig 4B**). These results indicate that for dim signals, temporal rank filters produce a broader range of estimates of pixel intensity than frame averaging, even when accounting for the higher pixel intensity values produced by the rank filter. However, the boost in sample size associated with switching from a temporal (1x1xn) rank filter to a spatiotemporal (3x3xn) mitigates this variation and produces estimates with less fluctuation than frame averaging.

**Fig 5:**
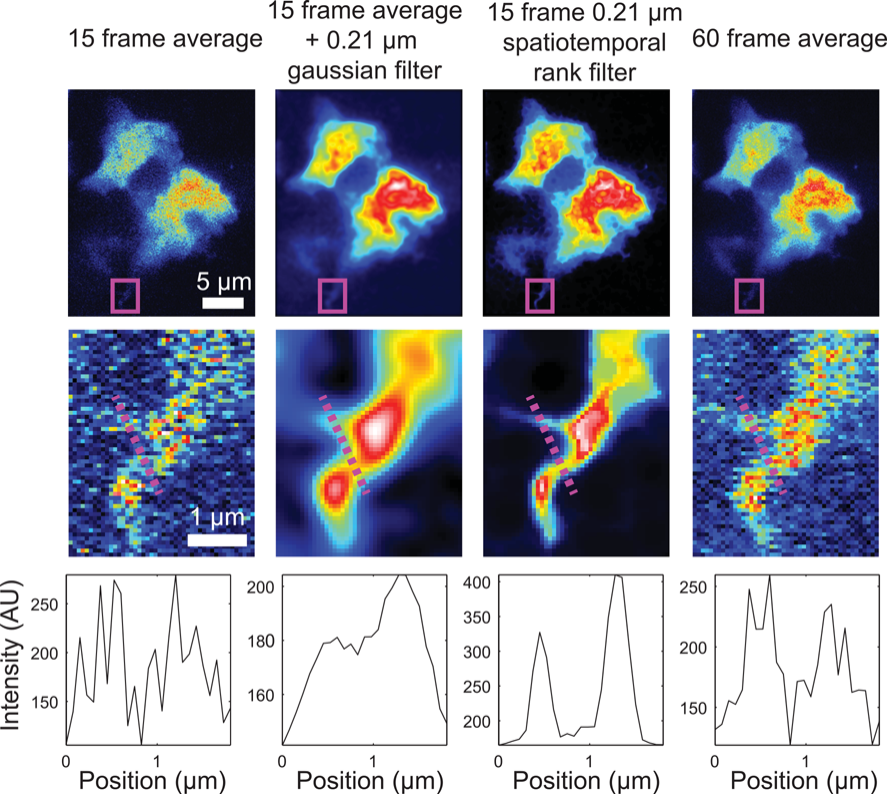
Comparison of spatiotemporal rank filter with frame averaging and frame averaging followed by Gaussian filtering. Dendritic cell within the lymph node of an XCR1-Venus transgenic mouse. Digitally zoomed region shows that spatiotemporal rank filtering enables high-contrast visualization of a dendritic process without blurring its edges.

Finally, we sought to verify the utility of spatiotemporal rank filters to preserve fine image details. We collected a series of images of fluorescently labeled dendritic cells within the lymph node of an XCR1-Venus transgenic mouse. Spatiotemporal rank filtering was able to generate a clean image of dendritic processes while preserving the sharp edges that could clearly be seen from an image averaged from 4x as many frames, unlike frame averaging followed by Gaussian filtering, which blurred these details (**Fig 5**).

These images also demonstrate a potential pitfall of spatiotemporal rank filtering compared to frame averaging: the introduction of artifacts on the order of the size/shape of the spatial component of the filter. The spatial component of the filter used in **Fig 5**was approximately circular with a radius of 3 pixels, and the spatiotemporally rank filtered images show many local regions where groups of pixels with the same size and shape as the filter are homogenized in value. Thus, while increasing the spatial size of spatiotemporal rank filters produces pixel intensity estimates with lower error on average, image details comprised of spatial frequencies higher than the filter size should be interpreted with caution.

## Discussion

In addition to the popular median filter, the utility of the more general class of rank filters in image processing applications has been demonstrated extensively [15,16,20,21,25,29]. Here, we have described the spatiotemporal rank filter as an alternative method to frame averaging widely used in 2P-LSM that is better suited to filtering out the noise present in this modality of imaging. In practice, spatiotemporal rank filtering allows for improvements in temporal and/or spatial resolution, the generation of signal that is easier to detect and analyze, reduced excitation power, or some combination of these. We have shown here that this method can be used on images at multiple levels of depth, a crucial characteristic for its use in imaging 3D volumes. Applying the spatiotemporal rank filter rather than frame averaging in our experience has consistently yielded at least 4x improvements in speed of acquisition. The rank filter is well characterized in the signal processing literature, simple, and requires no special hardware to implement on existing instruments. Fast algorithms have already been described for its implementation [25,30].

The choice of parameters is a crucial consideration for the implementation of spatiotemporal rank filters. The optimal rank parameter depends on the relative levels of signal and noise. As signals grow stronger, the tails of their pixel distributions grow longer and the optimal rank tends more towards 0.5 (a median filter) [29]. For a weaker signal, a smaller proportion of pixels will contain signal and higher ranks must be used to avoid erasing signals. We have found a good rule of thumb is to find the rank value at the filter begins to fail to attenuate background pixels, and use a value slightly lower than this. This choice will eliminate noise while preserving the weakest fluorescent signals that are distinguishable above background. Importantly, the optimal rank parameter will likely drop with increased depth of imaging over large volumes, as out of focus fluorescence grows stronger relative to dim signals [12]. The nonlinear relationship of rank filtered intensity values compared to frame averaged values indicates that the former cannot be considered proportional to the concentration of fluorescent molecules. However, measuring fluorescence intensity in 3D 2P-LSM images is usually an intractable problem because of the lack of reliable methods to quantify the relative effects of scattering of the excitation beam and absorption of emitted fluorescence at different depths of imaging, both of which contribute to a loss of signal. So while rank filtering does distort the linearity of the relationship between fluorophore concentration and measured intensity, in practice this relationship does not hold across 3D volumes anyway. Still, rank filtering with the appropriate parameter can produce images that are much better suited for the typical uses of multiphoton images: visually identifying and computationally localizing objects such as cells within tissues.

While rank filtering is capable of recovering high quality images from a much smaller number of input frames compared to averaging, there are several important considerations in its implementation. As the edges of the bead PSFs under rank filtering compared to averaging show (**Fig 3E**), the former has the potential to attenuate weaker signals, depending on the choice of rank parameter. In this case of a single fluorescent bead, attenuation of the PSF edges yield an image that more closely resembles the underlying object (a point source 1 pixel in diameter). However, unlike a linear filter, for the nonlinear rank filter this outcome cannot be extrapolated to collections of overlapping PSFs that would occur in a biological sample. Indeed, owing to the reliance of nonlinear filters on the probability distributions of their inputs, it is challenging to generalize the behavior of the rank filter across all situations. For example, while rank filters are excellent at preserving sharp edges essential for human perception and computational analysis (as shown in **Fig 1C, Fig 3E, Fig 5**), the exact shape of these edges is a complex function of the image shape and SNR, as well as the size and shape of the filter [31,32].

In general, there is an implicit tradeoff in choosing the size of the spatial component of rank filters: larger spatial windows reduce the normalized mean squared error of pixel intensity estimates, but have the potential for introducing spatial artifacts or the loss of details smaller than the window size. On the other hand, smaller spatial windows require compensatory increases in the number of frames used, or they will increase stochastic fluctuations in input values below the threshold that enables recovery of signal from background noise. In the latter case, even with an appropriately chosen rank value, weaker parts of signals may be stochastically attenuated (such as parts of the cell edges in the 5 frame spatiotemporal rank filtered image compared to the 30 frame averaged image in **Fig 1C**). For applications in which the minimization of the potential for spatial artifacts is important, filters with smaller spatial windows should be utilized. In addition, more complex extensions of rank filters can be designed to preserve specific types of morphological features [25,33].

In spite of these limitations and tradeoffs, we have demonstrated here that spatiotemporal rank filtering can significantly improve the performance of 2P-LSM in its common application of *in vivo* observation of dynamic biological systems on the cellular and subcellular level. In spite of their potential for inducing changes in the shape of edges, they nonetheless produce brighter and higher contrast images of T-cells over a large 3D volume (**Fig 1**) suitable for tracking of cell movements over time. Similarly, the essential features of dendritic process can be captured with a much reduced use of photons using rank filtering (**Fig 5**). The sharpened edges of this process may even better reflect the shape of the underlying object before its convolution by the imaging system. However, without knowledge of shape of the PSF at the particular depth of imaging, quantitative measurements of morphology or intensity of these features must be interpreted cautiously, if at all.

In spite of these considerations, with a filter neighborhood no larger than the highest spatial frequencies our system was able to collect according to its optical resolution, spatiotemporal rank filtering still significantly outperformed frame averaging in a mean squared error sense for the recovery of dim signals. For experiments that do not require the system’s full optical resolution (such as cell tracking), larger spatial neighborhoods can be used to further improve the quality of the lower resolution portion of images.

In addition to replacing the relatively inefficient linear filtering operation used in frame averaging, a second advantage of the spatiotemporal rank filter used here is its combination of the in-acquisition and post-acquisition filtering operations into a single step. While spatial rank filters (i.e. without a temporal component) are useful in processing standard frame-averaged images, the loss of information about pixel distributions that occurs when they are averaged into a single value is detrimental to the performance of the filter. As the ease of storing 2-photon microscopy data continues to increase, it may be advantageous to save each individual frame at acquisition time, and adjust filter parameters after acquisition, much like contrast can be adjusted when viewing data.

## Conclusions

Overall, the spatiotemporal rank filter is a simple yet robust method of dramatically improving image quality, increasing acquisition speed, and decreasing phototoxicity when using 2P-LSM to image dynamic biological processes. It can be implemented without any additional hardware to enable new types of imaging experiments. In spite of its utility, we emphasize that there is ample room for future improvement, both as variants of the spatiotemporal rank filter itself and in the application of other types of nonlinear filters to raw 2P-LSM data.

## Materials and Methods

### Two-photon microscopy

Bead and lymph node data were collected on either a custom built resonant scanning two-photon or Nikon A1R resonant scanning two-photon microscope using a 20X water immersion 1.05NA or 60x water immersion 1.20NA (**Fig 5**) objective, respectively, at 30fps.

### Biological samples

All mice were bred and housed in specific pathogen–free housing and in strict accordance with the guidelines of the Laboratory Animal Resource Center of the University of California, San Francisco (UCSF). Experiments were approved by the UCSF Institutional Animal Care and Use Committee (Approval #AN106779-02B). CD8+ T cells were isolated from lymph nodes of a C57BL/6 mouse using a negative CD8+ Isolation kit (Stemcell Technologies). Isolated cells were then labeled with Violet Proliferation Dye 450 (VPD-450) (BD Biosciences) according to the manufacturer’s protocol. 2x10^6^ labeled cells were adoptively transferred intravenously into a wild type C57 B6 mouse. After waiting 24 hours to allow cells to home to lymphoid organs, the mouse was euthanized by CO_2_ inhalation followed by cervical dislocation, and an inguinal lymph node was explanted. 200 μm Z-stacks were collected extending from the lymph node cortex, with 30 individual scans at each Z-slice for later processing into frame averaged or rank filtered images. The excitation laser was tuned to 810 nm, and its power was automatically increased with depth in order to compensate for scattering. XCR1-Venus dendritic cells were imaged in an explanted inguinal lymph node just below the cortex at the node’s center.

### Bead images

175 nm diameter PS-SPECK yellow-green beads were diluted 1:1000 and mounted on L-Lysine treated slides to prevent the beads from drifting. 1000 consecutive images were taken at 860 nm at a laser power low enough that 5–10 frames needed to be averaged to visually distinguish individual beads. The yellow emission channel (568LP dichroic with 542/27 emission filer) was used for all subsequent analysis. A dark image was taken with detectors turned on but the excitation laser turned off, and the 50^th^ percentile of the pixels in this image was subtracted from all bead images prior to analysis in Matlab. Signal to background ratio was calculated as the mean value of pixels lying within a mask for signal versus those lying within a mask for background. These masks were thresholded from the 1000-frame averaged images, with spatial frequencies higher than the Abbe limit of the system filtered out. Each bootstrapping sampling distribution was composed of 10^5^ samples. Normalized mean square error was defined as:

$$\frac{1}{\text{n estimates}}=\sum_{\text{all estimates}}{\frac{\left(\text{estimate - population value}\right)}{{\left(\text{population value}\right)}^{2}}}^{2}$$

### Spatiotemporal rank filtering

All spatiotemporal rank filter images were created with pixelated approximations of circular windows. After being spatiotemporally rank filtered, the resulting images were filtered again with the same spatial window and rank parameter of (1 –initial rank parameter) to reverse the extension of edges as described in [25].
